# Plasma apolipoprotein concentrations and incident diabetes in subjects with prediabetes

**DOI:** 10.1186/s12933-022-01452-5

**Published:** 2022-02-07

**Authors:** Mikaël Croyal, Matthieu Wargny, Kevin Chemello, Chloé Chevalier, Valentin Blanchard, Edith Bigot-Corbel, Gilles Lambert, Cédric Le May, Samy Hadjadj, Bertrand Cariou

**Affiliations:** 1 Nantes Université, CHU Nantes, CNRS, INSERM, l’institut du Thorax, 8 quai Moncousu, 44000 Nantes, France; 2 Nantes Université, CHU Nantes, Inserm, CNRS, SFR Santé, Inserm UMS 016, CNRS UMS 3556, 44000 Nantes, France; 3 CRNH-Ouest Mass Spectrometry Core Facility, 44000 Nantes, France; 4 CHU de Nantes, INSERM CIC 1413, Pôle Hospitalo-Universitaire 11: Santé Publique, Clinique des données, Nantes, France; 5 Université de La Réunion, INSERM UMR 1188 DéTROI, Sainte-Clotilde, France; 6 Departments of Medicine, Centre for Heart Lung Innovation, Providence Healthcare Research Institute, St. Paul’s Hospital, University of British Columbia, Vancouver, Canada; 7 Department of Biochemistry, CHU Nantes, G et R Laënnec Hospital, Bd Jacques Monod, Nantes, France

**Keywords:** New-onset diabetes, Type 2 diabetes, Apolipoproteins, Apolipoprotein E, IT-Diab study

## Abstract

**Background:**

The identification of circulating biomarkers associated with the risk of type 2 diabetes (T2D) is useful for improving the current prevention strategies in the most at-risk patients. Here, we aimed to investigate the association of plasma apolipoprotein concentrations in prediabetes subjects with the incidence of new-onset T2D during follow-up.

**Methods:**

In the IT-DIAB prospective study, 307 participants with impaired fasting glucose levels (fasting plasma glucose [FPG]: 110–125 mg/dL) were followed yearly for 5 years. The onset of T2D was defined as a first FPG value ≥ 126 mg/dL during follow-up. Apolipoprotein (apo)A-I, A-II, A-IV, B100, C-I, C-II, C-III, C-IV, D, E, F, H, J, L1, M, and (a) plasma concentrations were determined by mass spectrometry. Correlations between apolipoproteins and metabolic parameters at baseline were assessed by Spearman’s coefficients. Kaplan–Meier curves were drawn using a ternary approach based on terciles and incident T2D. The association between plasma apolipoproteins concentrations and the incidence of T2D was determined using Cox proportional-hazards models.

**Results:**

During a median follow-up of 5-year, 115 participants (37.5%) developed T2D. After adjustment for age, sex, body mass index, FPG, HbA_1c_, and statin use, the plasma levels of apoC-I, apoC-II, apoC-III, apoE, apoF, apoH, apoJ, and apoL1 were positively associated with a high risk for T2D. After further adjustment for plasma triglycerides, only apoE (1 SD natural-log-transformed hazard ratio: 1.28 [95% confidence interval: 1.06; 1.54]; *p* = 0.010), apoF (1.22 [1.01; 1.48]; *p* = 0.037), apoJ (1.24 [1.03; 1.49]; *p* = 0.024), and apoL1 (1.26 [1.05; 1.52]; *p* = 0.014) remained significantly associated with the onset of T2D. Kaplan–Meier survival curves also showed that the lower third of plasma apoE levels (< 5.97 mg/dL) was significantly associated with a lower risk of conversion to T2D (log-rank test, *p* = 0.002) compared to the middle and upper thirds.

**Conclusions:**

The plasma apoE levels are positively associated with the risk of T2D in prediabetes subjects, independently of traditional risk factors. The possible associations of apoF, apoJ, and apoL1 with T2D risk also pave the way for further investigations.

*Trial registration* This trial was registered at clinicaltrials.gov as NCT01218061 and NCT01432509

**Supplementary Information:**

The online version contains supplementary material available at 10.1186/s12933-022-01452-5.

## Background

Currently, 463 million people are living with diabetes worldwide [1], and recent predictions estimate that the number of affected people may increase to 578 million by the year 2030 [2]. Type 2 diabetes (T2D) is the predominant form of the disease and results from complex interactions between modifiable and unmodifiable risk factors. T2D is characterized by chronic hyperglycemia leading to serious tissue damage that reduces both the quality of life and life expectancy of affected individuals, especially by promoting cardiovascular and renal complications [3, 4]. Thus, T2D is considered as a major health burden worldwide and one of the most important modifiable cardiovascular risk factors [5]. Hence, the identification of early biomarkers associated with T2D risk is required for improving the current prevention strategies and for gaining a better understanding of the disease [6].

Abnormalities in lipid and lipoprotein metabolism have been identified as risk factors for T2D. Low concentrations of high-density lipoprotein cholesterol (HDL-C) and high concentrations of plasma triglycerides (TG) are associated with an increased risk of T2D, and they are usually preceded by the gradual onset of insulin resistance [7, 8]. Clinical trials also have reported that the statin-induced reduction of low-density lipoprotein cholesterol (LDL-C) slightly increases the risk of new-onset T2D [9–11]. Moreover, Mendelian randomization studies have shown that loss-of-function variants in the 3-hydroxy-3-methylglutaryl-CoA reductase (*HMGCR*) gene, the molecular target of statins, and in the proprotein convertase subtilisin/kexin type 9 (*PCSK9*) gene, a natural inhibitor of the LDL receptor, are associated with an increased risk of T2D [12]. In contrast, T2D prevalence is halved in patients with familial hypercholesterolemia, especially in carriers of the most severe LDL receptor mutations [13], suggesting a direct association between the prevalence of T2D and the upregulation of the LDL receptor pathway.

The metabolism of lipids and lipoproteins is dependent on apolipoproteins, a class of multifunctional proteins that govern the assembly of lipoprotein particles, maintain their structure, and direct their metabolism through binding to cell-surface receptors and regulating enzyme activity [14]. Several studies have demonstrated that the plasma levels of apolipoproteins better predict T2D than traditional plasma lipids, independently of classical risk factors [15–17]. Large cohort studies drawn from the general population have shown that the apolipoprotein (apo)B100-to-LDL-C, apoA-I-to-HDL-C, and apoA-II-to-HDL-C ratios are better predictors of worsening glycemia and incident T2D than LDL-C and HDL-C levels, respectively [15, 16]. More recently, another prospective population-based cohort study has revealed that the serum levels of apoC-III and the apoC-III-to-apoA-I ratio are strongly associated with incident T2D [7].

However, such studies only focused on major apolipoprotein species. Meanwhile, several reports have pointed out that other apolipoproteins (e.g., apoC-I, apoC-II, apoD, apoF, apoH, apoJ, apoL1, and apoM), whose metabolic functions are less understood, deserve further investigation [14, 18]. In this ancillary analysis from the prospective IT-DIAB study, we investigated the association between plasma concentrations of 16 apolipoproteins determined by a validated mass-spectrometry multiplex assay [19] with the risk of new-onset T2D in individuals with prediabetes.

## Methods

### Study population

**Fig. 1:**
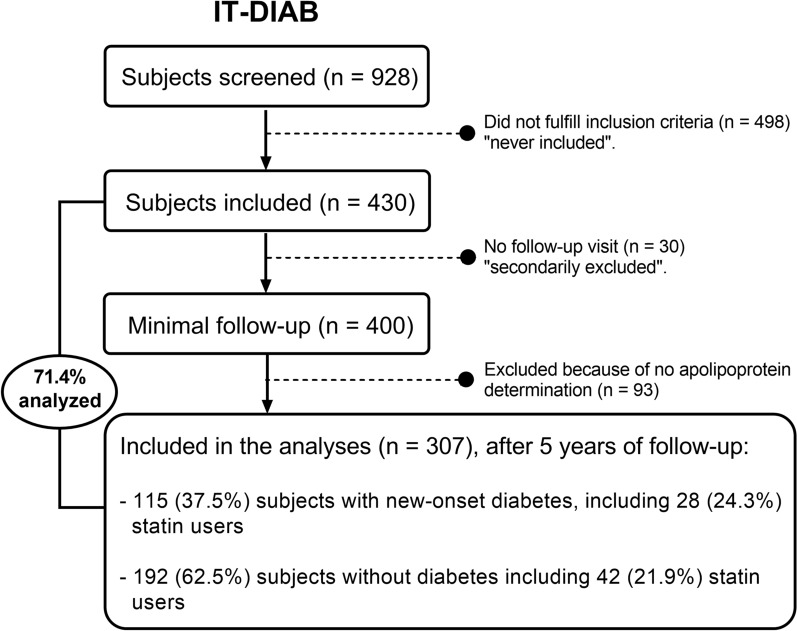
Flow chart of the IT-DIAB study

The IT-DIAB study (Innovation Thérapeutique-Diabète NCT01218061 & NCT01432509) is a 5-year prospective, observational study designed to identify new biomarkers of T2D risk in a population with prediabetes. Details on the study design, recruitment, and procedures have been reported elsewhere [20, 21]. The institutional ethics committee approved the protocol, and all of the reported investigations were carried out in accordance with the principles of the Declaration of Helsinki as revised in 2008. All subjects underwent a baseline visit between June 2010 and February 2013, including a medical interview, signing of the informed consent, self-administered questionnaire of diabetes risk score, physical examination (including body weight, height, waist, and hip circumference measurements), and blood sampling. Patients without a history of diabetes and with an impaired fasting glucose (IFG) level, defined as a fasting plasma glucose (FPG) level of 110–125 mg/dL (according to the World Health Organization classification), were eligible for the IT-DIAB study. The main exclusion criteria were a history of treatment with antidiabetic agents or insulin (with the exception of gestational diabetes), severe coagulation disorder or thrombocytopenia (platelet level < 100,000/mm^3^), severe renal insufficiency (defined using the Modification of Diet in Renal Disease equation as an estimated glomerular filtration rate < 30 mL/min/1.73 m^2^), severe liver impairment (prothrombin ratio < 50%), severe psychiatric disorder, alcohol abuse (estimated to be > 30 g/day), subject’s opposition, and an inability to participate for at least 5 years in the study. For the present analysis, 307 subjects with at least one follow-up visit were considered (Fig. 1).

### Follow-up and conversion to new-onset diabetes

The end of the follow-up period occurred at the fifth yearly visit, or prematurely if the subject met one of the following criteria: subject withdrawal or lost to follow-up, inappropriate prescription of an antidiabetic agent, bariatric surgery, or death. New-onset diabetes was defined as a FPG value ≥ 126 mg/dL and/or a plasma glucose concentration ≥ 200 mg/dL after a 2-h 75-g oral glucose tolerance test.

### Biochemical analysis

During the baseline visit, peripheral venous blood samples were obtained in the morning after an overnight fast for biological analyses. Standard biological analyses included FPG, glycated hemoglobin (HbA_1c_), and the lipid profile (total cholesterol (TC), HDL-C, and TG). LDL-C was calculated using the Friedewald equation, and non-HDL-C was calculated as TC minus HDL-C. Frozen heparinized plasma was used for the insulin measurement by an electro-chemiluminescent enzyme immunoassay (ECLIA) using the Cobas e-automated clinical analyzer system (Roche Diagnostics, Meylan, France). Plasma high-molecular-weight adiponectin levels were measured by ECLIA on the Lumipulse G600 automated clinical analyzer system (Fujirebio, Les Ulis, France). Homeostasis model assessments of insulin resistance (HOMA-IR) and beta-cell function (HOMA-β) were defined according to the equations proposed by Matthews et al. [22].

### Apolipoprotein measurements

Plasma apolipoproteins A-I, A-II, A-IV, B100, C-I, C-II, C-III, C-IV, D, E, F, H, J, L1, M, and (a) as well as apoE phenotyping were determined by liquid chromatography-tandem mass spectrometry, as described previously [19]. Briefly, the apolipoproteins were quantified in 40-µL aliquots (EDTA plasma) using trypsin proteolysis and the subsequent analysis of proteotypic peptides. The apoE phenotypes (E2/E3/E4) were determined using a combination of five proteolytic peptides, as described previously [19, 23]. The intra- and inter-assay variabilities did not exceed 9.4%.

### Statistical analysis

All categorical parameters were expressed as the number (%). All quantitative parameters were expressed as the mean ± standard deviation (SD) or, when the distribution was considered as skewed, median [25th percentile; 75th percentile]. The correlation between the baseline characteristics was studied as a cross-sectional study. Spearman’s rank correlation coefficients were calculated between plasma apolipoprotein concentrations and the clinical characteristics, glucose homeostasis, and lipid profile. The associations between the plasma lipids, plasma apolipoproteins, and final diabetic status (no diabetes vs. new-onset diabetes) were first analyzed using the univariate logistic regression model. Kaplan–Meier curves were drawn using a ternary approach based on terciles and the incidence of new-onset diabetes as the event of interest. Then, we used univariate and multiple Cox regression models with the following adjustments: no adjustment (model 1); adjusted for baseline values of age, sex, body mass index (BMI), FPG and HbA_1c_ (model 2); model 2 and the use of statins or fibrates (model 3); and model 3 and TG (model 4). In the Cox analyses, we favored a systematic approach using log-natural transformation and standardization for all quantitative variables. The only exception was apoC-IV, which was used as a binary datum (detectable vs. not detectable). The proportional hazards assumption was tested based on Schoenfeld residuals. The first-order risk was set to 5%. The analyses were conducted on complete cases, without imputation. No correction was considered for multiple testing. All analyses were performed using R software, version 4.0.0 [24].

## Results

### Baseline characteristics of the study participants

The baseline characteristics of the study population are reported in Table 1. On average, the study population was middle aged (age: 57.3 ± 9.9 years old) and overweight (BMI: 29.8 ± 6.2 kg/m^2^). The median FPG was 115 [112; 119] mg/dL. Regarding lipid-lowering treatment, 83 participants (27%) declared to be on routine lipid lowering drug therapy (mostly under statins: 84%). The plasma HDL-C (53 ± 15 mg/dL), LDL-C (137 ± 36 mg/dL), and TG (119 [86; 167] mg/dL) levels were in the normal range (Table 2). During the 5-year follow-up (IQR 25–62 months, 262/307 patients (85.3%) until the 5th visit), 115 participants (37.5%) developed new-onset T2D.

**Table 1:** Baseline characteristics of the subjects according to their diabetes status at the end of the follow-up

Parameters	All (n = 307)	No diabetes (n_1_ = 192)	Diabetes (n_2_ = 115)	Available data (n_1_/n_2_)
Clinical data				
Age (y)	57.3 ± 9.9	57.2 ± 10.4	57.5 ± 9.0	192/115
Sex (female)	92 (30.0%)	54 (28.1%)	38 (33.0%)	192/115
BMI (kg/m^2^)	29.8 ± 6.2	28.8 ± 5.9	31.4 ± 6.3	192/115
Waist circumference (cm)	99 ± 14.6	96.8 ± 14.0	102.7 ± 14.9	191/114
Hip circumference (cm)	105.2 ± 13.1	103.4 ± 11.2	108.2 ± 15.3	188/112
Waist/hip circumference ratio	0.94 ± 0.09	0.93 ± 0.09	0.95 ± 0.09	188/112
Statin use	70 (22.8%)	42 (21.9%)	28 (24.3%)	192/115
Fibrate use	11 (3.6%)	5 (2.6%)	6 (5.2%)	192/115
Ezetimibe use	2/307 (0.7%)	0/192 (0%)	2/115 (1.7%)	192/115
Glucose homeostasis				
FPG (mg/dL)	115 [112; 119]	114 [112; 118]	116 [113; 121]	192/115
HbA_1c_ (%)	5.9 [5.6; 6.2]	5.8 [5.5; 6.0]	6.0 [5.8; 6.3]	191/115
Insulin (mIU/L)	14.1 ± 9.1	12.4 ± 8.1	17.1 ± 9.9	182/103
HOMA-IR	4.02 ± 2.69	3.46 ± 2.39	5.01 ± 2.90	182/103
HOMA-β (%)	100.6 ± 65.9	92.9 ± 61.5	114.1 ± 71.3	182/103
Adiponectin (µg/L)	3.70 ± 2.20	3.95 ± 2.30	3.28 ± 1.91	183/103

Categorical parameters are expressed as the population size (%). Quantitative parameters are expressed as the mean ± SD in the case of Gaussian distribution, otherwise as the median [25th percentile; 75th percentile]. BMI: body mass index; FPG: fasting plasma glucose; HOMA-IR/**-**β: homeostasis model assessment of insulin resistance/β-cell function

**Table 2:** Baseline plasma concentrations of apolipoproteins and lipids and the risk of diabetes during follow-up (univariate logistic regression models)

Apolipoproteins	All (n = 307)	No diabetes (n_1_ = 192)	New-onset diabetes (n_2_ = 115)	OR (95% CI)	*p*-value	Data available (n_1_/n_2_)
Total cholesterol (mg/dL)	217 ± 40	218 ± 40	215 ± 40	0.93 (0.74–1.17)	0.5486	191/114
Triglycerides (mg/dL)	119 [86; 167]	108 [83; 154]	137 [94; 173]	1.33 (1.05–1.69)	0.0199*	191/113
LDL-C (mg/dL)	137 ± 36	138 ± 37	136 ± 35	0.96 (0.76–1.21)	0.7202	188/112
HDL-C (mg/dL)	53 ± 15	55 ± 16	50 ± 13	0.79 (0.62–1.00)	0.0461*	191/113
Non-HDL-C (mg/dL)	164 ± 41	164 ± 42	164 ± 38	1.03 (0.82–1.31)	0.7794	191/113
ApoA-I (mg/dL)	123 [106; 143]	124 [106; 144]	122 [106; 142]	0.93 (0.74–1.17)	0.5269	192/115
ApoA-II (mg/dL)	22.7 [19.1; 27.9]	22.3 [19.1; 27.8]	23.4 [19.2; 28.1]	1.08 (0.86–1.36)	0.5158	192/115
ApoA-IV (mg/dL)	8.4 [6.3; 10.8]	8.4 [6.4; 10.8]	8.5 [5.9; 10.7]	0.99 (0.79–1.25)	0.9469	192/115
ApoB100 (mg/dL)	56.0 [43.5; 71.2]	56.5 [45.0; 71.7]	53.7 [41.9; 68.9]	0.94 (0.75–1.19)	0.6266	192/115
ApoC-I (mg/dL)	1.12 [0.92; 1.44]	1.12 [0.89; 1.38]	1.13 [0.94; 1.53]	1.17 (0.93–1.47)	0.1766	192/115
ApoC-II (mg/dL)	2.63 [1.92; 3.48]	2.46 [1.88; 3.48]	2.84 [2.08; 3.62]	1.35 (1.06–1.70)	0.0133*	192/115
ApoC-III (mg/dL)	6.22 [4.91; 8.01]	6.04 [4.88; 7.51]	6.73 [5.23; 9.22]	1.27 (1.01–1.61)	0.0430*	192/115
ApoC-IV (mg/dL)	0.0 [0.0; 0.12]	0.0 [0.0; 0.13]	0.0 [0.0; 0.11]	1.03 (0.82–1.29)	0.8204	192/115
ApoD (mg/dL)	2.88 [2.37; 3.55]	2.98 [2.43; 3.65]	2.78 [2.35; 3.30]	0.86 (0.68–1.09)	0.2026	192/115
ApoE (mg/dL)	6.96 [5.61; 8.61]	6.45 [5.44; 8.35]	7.61 [6.11; 9.14]	1.31 (1.04–1.66)	0.0232*	192/115
ApoF (mg/dL)	0.82 [0.46; 1.20]	0.79 [0.46; 1.14]	0.90 [0.48; 1.36]	1.22 (0.96–1.53)	0.0994	192/115
ApoH (mg/dL)	4.66 [3.95; 5.52]	4.47 [3.88; 5.42]	4.77 [4.14; 5.94]	1.25 (0.98–1.58)	0.0670	192/115
ApoJ (mg/dL)	7.96 [6.66; 9.44]	7.83 [6.66; 9.26]	8.43 [6.65; 9.77]	1.26 (1.00–1.60)	0.0502	192/115
ApoL1 (mg/dL)	1.12 [0.92; 1.35]	1.09 [0.91; 1.28]	1.20 [0.95; 1.52]	1.43 (1.13–1.81)	0.0032**	192/115
ApoM (mg/dL)	1.85 [1.50; 2.32]	1.85 [1.55; 2.33]	1.85 [1.45; 2.28]	0.91 (0.71–1.16)	0.4447	192/115
Apo(a) (nmol/L)	22.7 [0.0; 62.4]	22.8 [0.0; 60.9]	21.9 [0.0; 64.1]	1.02 (0.81–1.29)	0.8685	192/115

Quantitative parameters are expressed as the mean ± SD in the case of Gaussian distribution, otherwise as the median [25th percentile; 75th percentile]. OR are calculated per 1 SD using univariate logistic regression models. The associated *p*-value is calculated using Wald test (*p < 0.05; **p < 0.01). LDL-C: low-density lipoprotein cholesterol; HDL-C: high-density lipoprotein cholesterol; OR: odds ratio; CI: confidence interval

### Baseline concentrations of plasma lipids and apolipoproteins

The baseline concentrations of plasma lipids and apolipoproteins, according to the diabetes status (diabetes vs. no diabetes) at the end of the individual follow-ups, are reported in Table 2. The plasma TG concentrations were higher in the participants who developed T2D (146 ± 73 vs. 129 ± 76 mg/dL; odds ratios (OR) [95% confidence interval (CI)] 1.33 [1.05–1.69], *p* = 0.020), whereas the HDL-C levels were significantly lower (50 ± 13 vs. 55 ± 16 mg/dL; OR = 0.79 [0.62–1.00], *p* = 0.046) compared to those who did not develop T2D. No significant difference was found for baseline TC, LDL-C, or non-HDL-C concentrations according to the final diabetes status. In addition, univariate analyses showed no statistically significant difference in the baseline levels of apoA-I, apoA-II, apoA-IV, apoB100, apoC-I, apoC-IV, apoD, apoF, apoH, apoJ, apoM, or apo(a) between the participants who developed new-onset T2D during follow-up and the others. In contrast, the baseline concentrations of some apolipoproteins were significantly higher in subjects with new-onset T2D vs. the others: apoC-II (2.84 [2.08; 3.62] vs. 2.46 [1.88; 3.48] mg/dL, OR = 1.35 [1.06–1.70], *p* = 0.013); apoC-III (6.73 [5.23; 9.22] vs. 6.04 [4.88; 7.51] mg/dL, OR = 1.27 [1.01–1.61], *p* = 0.043), apoE (7.61 [6.11; 9.14] vs. 6.45 [5.44; 8.35] mg/dL, OR = 1.31 [1.04–1.66], *p* = 0.023), and apoL1 (1.20 [0.95; 1.52] vs. 1.09 [0.91; 1.28] mg/dL, OR = 1.43 [1.13–1.81], *p* = 0.0032). Interestingly, additional univariate analyses carried out after the exclusion of subjects on statin therapy at baseline (n = 237 subjects remaining) showed similar results (Additional file 1: Table S1).

### Associations between plasma apolipoprotein levels and metabolic parameters

**Fig. 2:**
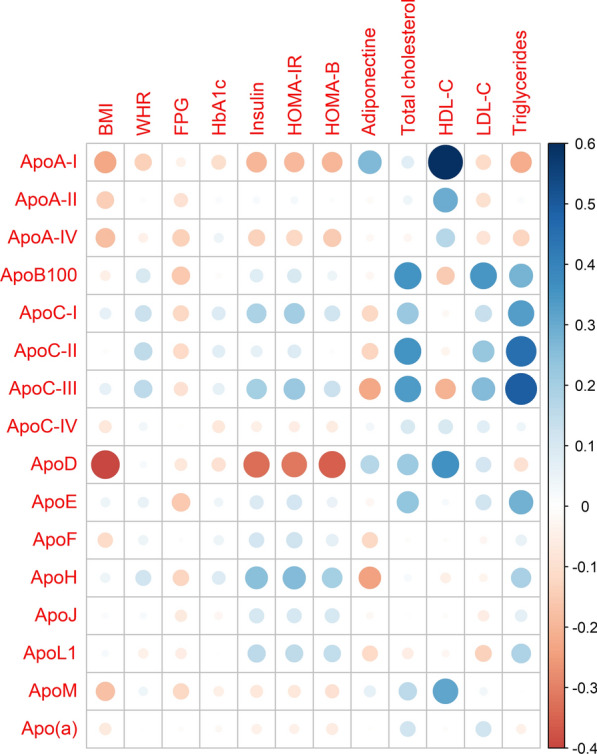
Spearman correlations between plasma apolipoprotein concentrations and biochemical parameters in IT-DIAB participants without statin treatment at baseline. BMI: body mass index; FPG: Fasting Plasma Glucose; HOMA-β: Homeostatic Model Assessment of Insulin beta-cell function; HOMA-IR: Homeostatic Model Assessment of Insulin Resistance; LDL-C: low-density lipoprotein cholesterol; HDL-C: high-density lipoprotein cholesterol; WHR: Waist/hip circumference ratio

Spearman’s rank correlation coefficients were calculated between the plasma apolipoproteins and the metabolic parameters at baseline for participants without lipid-lowering therapy (Fig. 2, Additional file 1: Table S2). As anticipated, the plasma apolipoprotein concentrations were correlated with the plasma lipid levels. Plasma apoA-I, apoA-II, apoA-IV, apoD, and apoM were positively correlated with HDL-C (Spearman’s coefficient [R] = 0.16–0.60, *p* < 0.05), whereas plasma apoC-I, apoC-II, apoC-III, apoE, and apoL1 were positively correlated with plasma TG (R = 0.22–0.53, *p* < 0.05), and plasma apoB100 was correlated with LDL-C (R = 0.38, *p* < 0.0001). In addition, the HDL-related apolipoproteins (apoA-I, apoA-II, apoA-IV, apoD, and apoM) were negatively correlated with the BMI (R = − 0.17 to − 0.42, *p* < 0.05). None of the plasma apolipoproteins were associated with HbA_1c_, and only plasma apoB100 was negatively correlated with FPG (R = − 0.15, *p* = 0.026). The HDL-related apolipoproteins (apoA-I, apoA-IV, and apoD) were negatively correlated (R = − 0.14 to − 0.34, *p* < 0.05), while the TG-rich lipoprotein-related apolipoproteins (apoC-I and apoC-III) were positively correlated with the fasting plasma insulin level (R = 0.19–0.21, *p* < 0.01). Moreover, several plasma apolipoproteins were significantly correlated with insulin resistance (HOMA-IR) (apoC-I and apoC-III; R = 0.21 and 0.23, respectively, *p* < 0.001) and insulin secretion (HOMA-β) indexes (apoA-IV and apoJ; R = − 0.16 and 0.13, respectively, *p* < 0.05), or both (apoA-I, apoD, apoH, and apoL1, R = − 0.37–0.15; *p* < 0.05).

### Plasma apolipoprotein levels and risk of T2D during follow-up

**Fig. 3:**
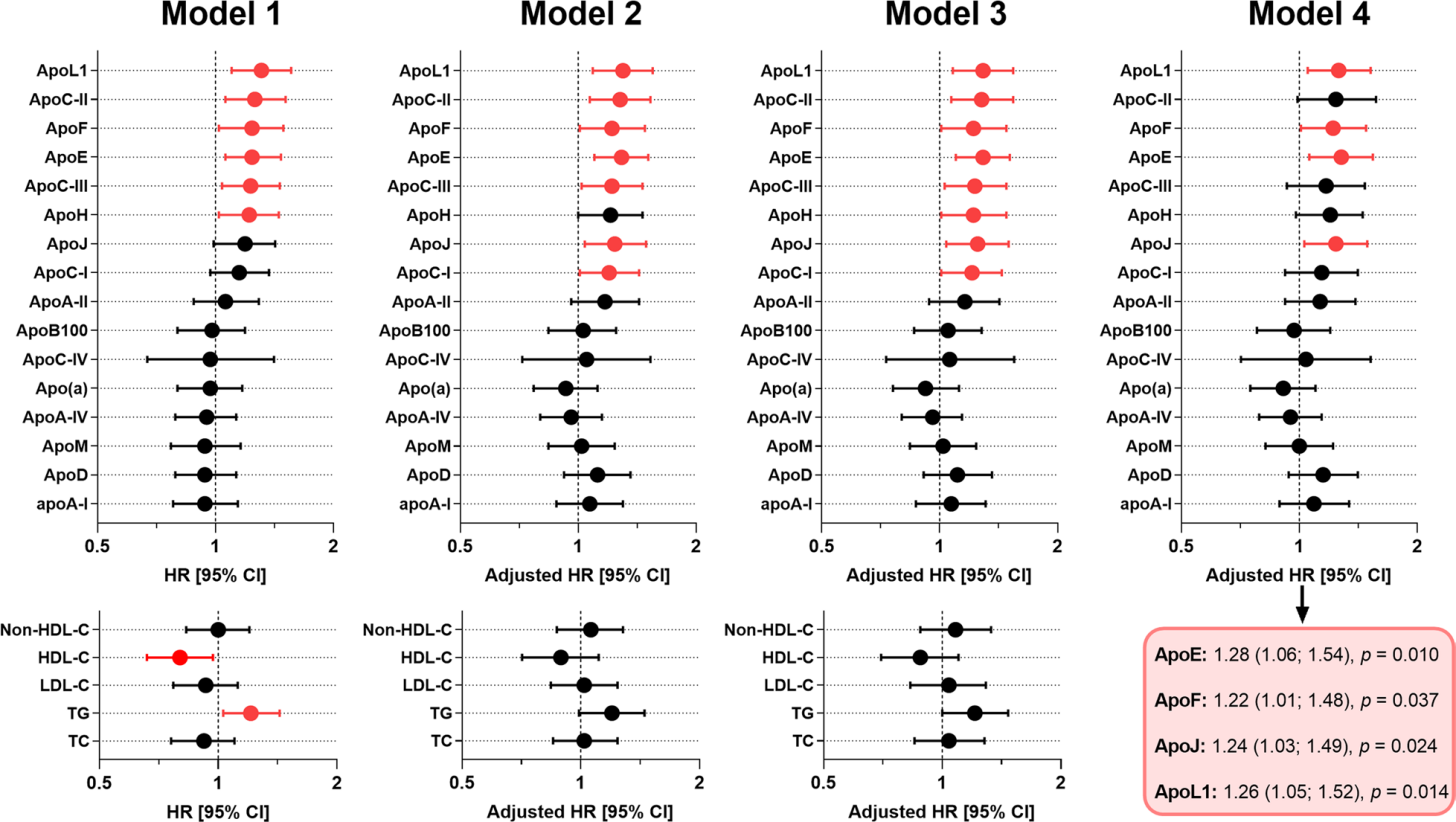
Association between plasma apolipoproteins and plasma lipids at baseline and the incidence of new-onset diabetes during follow-up (Cox models based on the proportional hazards assumption). Hazard ratios (HRs) are calculated per 1 SD after natural-log transformation. Red dots indicate significance with *p* < 0.05. Model 1: not adjusted (univariate). Model 2: adjusted for baseline values of age, sex, body mass index, fasting plasma glucose, and HbA_1c_. Model 3: model 2 with additional adjustment for the use of statins or fibrates. Model 4: model 3 with additional adjustment for triglycerides (TG). TC: total cholesterol; LDL-C: low-density lipoprotein cholesterol; HDL-C: high-density lipoprotein cholesterol

**Fig. 4:**
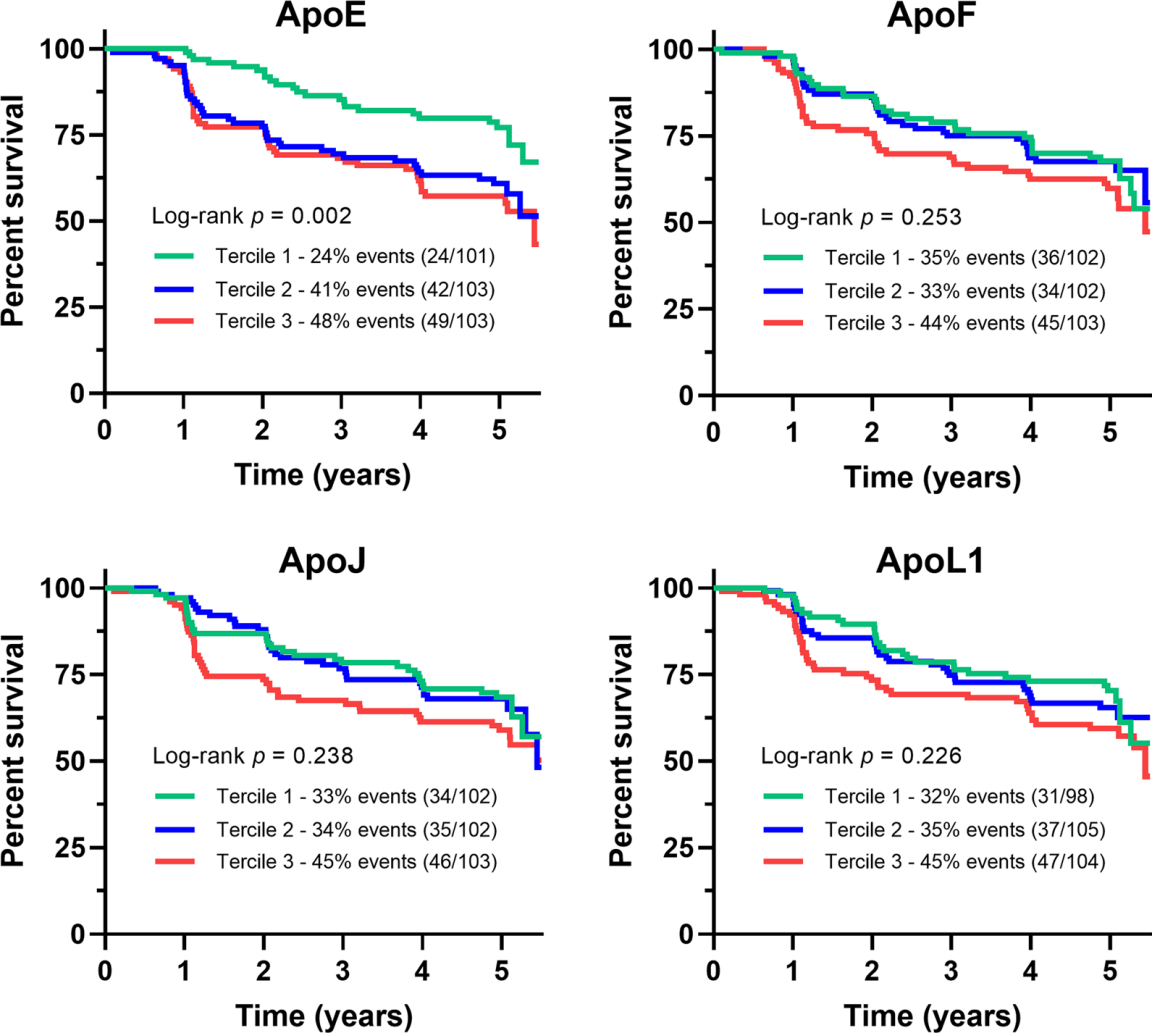
Survival curves for new-onset diabetes in the IT-DIAB cohort according to the baseline apoE, apoF, apoJ, and apoL1 plasma concentrations. The groups were created according to terciles. ApoE, tercile 1: 5.97 mg/dL; tercile 2: 8.06 mg/dL. ApoF, tercile 1: 0.63 mg/dL; tercile 2: 1.08 mg/dL. ApoJ, tercile 1: 7.07 mg/dL; tercile 2: 8.91 mg/dL. ApoL1, tercile 1: 0.96 mg/dL; tercile 2: 1.25 mg/dL

Results of the univariate and multivariable Cox model analyses are depicted in Fig. 3 and detailed in Additional file 1: Table S3. The baseline plasma concentrations of apoA-I, apoA-II, apoA-IV, apoB100, apoC-IV, apoD, apoM, apo(a), TC, LDL-C, and non-HDL-C were not associated with the onset of T2D during follow-up, both before (model 1) and after multiple adjustments (models 2–4). In contrast, before any adjustment, the baseline plasma concentrations of other apolipoproteins were associated with the conversion to T2D: apoC-II (hazard ratio (HR) per 1 SD [95% CI] 1.26 [1.06; 1.51], *p* = 0.010), apoC-III (HR = 1.23 [1.04; 1.46], *p* = 0.019), apoE (HR = 1.24 [1.06; 1.47], *p* = 0.009), apoF (HR = 1.24 [1.02; 1.49], *p* = 0.029), apoH (HR = 1.22 [1.02; 1.45], *p* = 0.029), apoL1 (HR = 1.31 [1.10; 1.56], *p* = 0.003). Furthermore, low HDL-C concentrations (HR per 1 SD = 0.80 [0.66; 0.97], *p* = 0.025) and high TG concentrations (HR per 1 SD = 1.21 [1.03; 1.43], *p* = 0.024) were associated with T2D risk. For HDL-C, plasma TG, and plasma apoH, such an association with new-onset diabetes did not remain after adjustment for sex, age, BMI, FPG, and HbA_1c_ (model 2). After additional adjustment for lipid-lowering therapy (model 3), the baseline plasma concentrations of apoC-I, apoC-II, apoC-III, apoE, apoF, apoH, apoJ, and apoL1 were significantly associated with the conversion to T2D; but only baseline apoE, apoF, apoJ, and L1 remained significant in the last model with additional adjustment for TG (HR: 1.28 [1.06; 1.54], *p* = 0.010; 1.22 [1.01; 1.48], *p* = 0.037; 1.24 [1.03; 1.49], *p* = 0.024; 1.26 [1.05; 1.52], *p* = 0.014; respectively). The Kaplan–Meier survival curves (Fig. 4) showed that the plasma concentrations of apoF, apoJ, and apoL1 (classified as terciles) were not significantly associated with the risk of conversion to T2D. In contrast, lower plasma concentrations of apoE (tercile 1) at baseline were significantly associated with a reduced risk of T2D (log-rank test, *p* = 0.002). Of note, no significant differences were found for the apoE phenotype distribution (apoE2/E3/E4) according to the final diabetes status of the participants at the final visit (*p* = 0.67, Additional file 1: Table S4). Finally, no interaction was observed between sex and plasma apoE concentrations regarding the risk of new-onset T2D during follow-up (*p*-value for interaction ≥ 0.90 in all three adjustment models, data not shown).

## Discussion

The present study aimed to deeply investigate the association of the plasma concentrations of a broad panel of apolipoproteins with the risk of new-onset T2D in individuals with prediabetes. After multivariable adjustment for traditional risk factors for T2D, including plasma TG levels, only the plasma apoE, apoF, apoJ, and apoL1 concentrations remained positively and significantly associated with new-onset T2D. The Kaplan–Meier survival curves showed that lower plasma levels of apoE at baseline were associated with a lower occurrence of T2D during follow-up.

Several studies have previously assessed the link between plasma lipid parameters and the risk of T2D, highlighting that both high TG and low HDL-C levels are independent risk factors for new-onset T2D in different populations [25, 26]. Recently, a large study performed in 451,933 participants of the UK Biobank confirmed that both low HDL-C and apoA-I levels were significantly associated with the risk of new-onset diabetes after multivariable-adjusted regressions and two-sample Mendelian randomization analyses [27]. Although we also found such an inverse association between low HDL-C levels and the risk of new-onset T2D by univariate analysis, it did not remain after further adjustments for BMI and glucose homeostasis (FPG, HbA_1C_). Beyond classical plasma lipid parameters, only a few studies have investigated the associations between plasma apolipoprotein levels and the risk of T2D, and there are even fewer reports focusing on the target population of subjects with prediabetes [7, 15–17]. Moreover, most of these studies focused only on major structural species like apoA-I and apoA-II for HDL, and apoB100 for very-low-density lipoprotein (VLDL) and LDL. Interestingly, some of them showed that apolipoproteins can better predict the risk of T2D than classical plasma lipid parameters in the general population [15, 16].

Thanks to a high-throughput mass spectrometry-based assay, we were able to extend the analysis to 16 apolipoprotein species [19]. After adjustment for classical diabetes risk factors, glucose parameters (FPG, HbA_1C_), and plasma TG, we ultimately identified 4 apolipoproteins amongst 16 that remain significantly associated with new-onset T2D in individuals with prediabetes: apoE, apoF, apoJ, and apoL1. With a similar approach, another prospective population-based cohort study also extended the analysis of apolipoproteins to apoA-I, apoC-III, apoD, and apoE [7]. After a long-term median follow-up of 13.5 years, 11.3% of individuals (110/971) developed T2D. While both apoC-III and apoE were significantly associated with the risk of T2D after adjustments for traditional risk factors, only apoC-III remained to be significantly associated with the risk of T2D after further adjustment for TG [7]. Our findings reinforce the hypothesis that apoC-III impacts glucose homeostasis through the regulation of TG and TG-rich lipoprotein metabolism. In accordance with such a role of apoC-III in the pathophysiology of T2D, a pilot study demonstrated that volanesorsen, an antisense oligonucleotide targeting *APOCIII* mRNA, improves insulin sensitivity and reduces HbA_1C_ in patients with T2D [28]. These promising findings need to be confirmed in larger randomized trials in patients with T2D and hypertriglyceridemia.

While apoE is a major human apolipoprotein that circulates primarily in association with HDL and VLDL [29], apoF, apoJ, and apoL1 are minor species in human plasma. ApoF circulates primarily as a component of HDL and LDL, and has been proposed to serve as a natural inhibitor of cholesterol ester transfer protein (CETP), with preferential inhibition of CETP activity with LDL [30]. There are no previous reports on the role of apoF in glucose metabolism. In our study, we did not find significant correlations between the plasma levels of apoF and metabolic parameters. However, it has been reported that pharmacological CETP inhibition with dalcetrapib or anacetrapib is associated with a reduced incidence of new-onset diabetes [31, 32]. ApoJ (also called clusterin) exists as multiple protein isoforms with contrasting properties. ApoJ circulates mainly as a component of HDL, and it has been implicated in a wide range of pathophysiological disorders [33]. In accordance with the role of apoJ in glucose homeostasis, apoJ can affect insulin signaling and inflammation [34]. Moreover, it has been reported that apoJ-knockout mice are insulin sensitive [35] and that the plasma levels of apoJ are elevated in obese subjects and positively associated with the BMI [36]. Finally, a Japanese study has found that a single nucleotide polymorphism of apoJ (rs22795590) is associated with a higher risk of T2D through both an increase in insulin resistance and an impairment of insulin secretion [37]. Surprisingly, we found that the apoJ levels were positively and significantly associated with HOMA-β but not with HOMA-IR, despite a trend (*p* = 0.062). It should be noted, however, that we were not able to discriminate the multiple isoforms of apoJ in the present study. ApoL1 also circulates primarily as a component of HDL [38]. Its plasma levels are correlated with plasma TG [39], a finding we also observed in our study population. The plasma levels of apoL1 have been reported to be increased in patients with metabolic syndrome, and an in vitro study has demonstrated that the insulin signaling pathway regulates both the synthesis and the secretion of apoL1 in hepatocytes [38]. In agreement with previous findings [38], we found a significant and positive correlation between apoL1 and HOMA-IR as well as an inverse correlation with plasma adiponectin, reinforcing its potential role in insulin resistance.

Finally, apoE was the apolipoprotein with the most robust association with new-onset T2D in our study. Indeed, we showed that a lower concentration (1st tercile) of apoE was associated with a reduced risk of new-onset T2D during the follow-up. ApoE predominantly acts as a ligand for the LDL receptor, the LDL receptor-related protein 1, and heparan sulfate proteoglycans, which mediate the clearance of triglyceride-rich lipoproteins and their remnants [23, 29]. A stable isotope kinetic study performed in obese patients has revealed that the VLDL and HDL apoE concentrations are increased in those with T2D as a result of increased production rates. In addition, HOMA-IR and HbA_1c_ were positively correlated with the VLDL apoE production rate in those patients [40]. While the plasma apoE concentrations were positively associated with both TG and LDL-C in our study, we failed to find significant correlations with markers of glucose homeostasis. A meta-analysis has shown that the *APOε2* allele is a moderate risk factor for T2D [41], in contrast to another study indicating that the *APOε4* allele is associated with T2D risk [42]. A recent study also has reported that the *ε2/ε2*, *ε3/ε4*, and *ε4/ε4 APOE* genotypes are associated with an increased risk of T2D [43]. However, due to the sample size of our study and the low frequency of the apoE2 and apoE4 isoforms, we were not able to clearly rule on these contradictory results.

Importantly, our results are in accordance with previous studies highlighting a relationship between plasma apoE levels and metabolic complications. As discussed above, Brahimaj et al*.* have reported a positive association between apoE and T2D risk, even if the statistical significance was lost after adjustment for plasma TG [7]. In the German population-based KORA F4/FF4 study (including people with prediabetes), Huth et al*.* also found that apoE is positively associated with new-onset diabetes beyond classical risk factors such as HbA_1c_, age, and sex [44]. Moreover, a plasma proteome analysis combined with Mendelian randomization analysis further suggested causal effects of apoE2 protein on the development of metabolic syndrome in two cohorts (KORA and HUNT_3_) [45].

Our study presents some limitations that must be acknowledged. Due to its exploratory approach, the alpha risk was set to 5% for each test without taking into account multiple testing. In addition, the relatively small size of our cohort limited the statistical power. Consequently, replication cohorts should be analyzed to assess the reproducibility of our results, especially for apoF, apoJ, and apoL1. Besides, our first objective was to investigate the possible associations between plasma concentrations of apolipoproteins and the risk of new-onset T2D in individuals with prediabetes beyond traditional risk markers. Hence, we did not consider this issue from the perspective of the added benefit of these biomarkers in prediction models (presenting area under the receiver operating characteristic (AUROC) or integrated discrimination index (IDI) calculation) as we did not aim to create a new predictive model of the risk of T2D. However, a major strength of our study is the simultaneous analysis of 16 apolipoproteins thanks to a multiplexed assay that has been carefully validated in terms of linearity, specificity, intra- and inter-assay precisions, accuracy, and stability after long-term storage and after multiple freeze/thaw cycles, making it suitable for cohort studies [19]. To the best of our knowledge, this study is the first to investigate so many apolipoproteins in a well-characterized at-risk population.

## Conclusions

Here, we showed that the plasma levels of apoE are associated with the occurrence of T2D in individuals with prediabetes, independently of traditional risk factors. Aside from the reported association between HDL-C or plasma TG with the risk of T2D in the general population, these findings strengthen the predictive value of plasma apolipoproteins in the development of metabolic diseases. However, additional studies are required to further determine the importance of apoE concentrations in triglyceride-rich lipoproteins and HDL for T2D risk assessment and for deciphering the underlying pathophysiological mechanisms. Furthermore, this study unraveled possible associations of minor plasma apolipoproteins (apoF, apoJ, and apoL1) with T2D, which paves the way for further investigations.

## Supplementary Information

**Additional file 1.** Additional Tables.

## Data Availability

The datasets associated with the current study are not publicly available but may be provided by the corresponding author on reasonable request.

## Abbreviations

Apo: Apolipoprotein
BMI: Body mass index
CETP: Cholesterol ester transfer protein
CI: Confidence interval
ECLIA: Electro-chemiluminescent enzyme immunoassay
FPG: Fasting plasma glucose
HbA_1c_: Glycated hemoglobin
HDL: High-density lipoprotein
HDL-C: HDL cholesterol
HMGCR: 3-Hydroxy-3-methylglutaryl-CoA reductase
HOMA-IR: Homeostasis model assessments of insulin resistance
HOMA-β: Homeostasis model assessments of beta-cell function
HR: Hazard ratio
IT-DIAB: Innovation Thérapeutique-Diabète
LDL: Low-density lipoprotein
LDL-C: LDL cholesterol
OR: Odds ratio
PCSK9: Proprotein convertase subtilisin/kexin type 9
SD: Standard deviation
T2D: Type 2 diabetes
TC: Total cholesterol
TG: Triglycerides
VLDL: Very-low-density lipoprotein
