# Supplementary material for: Plasma apolipoprotein concentrations and incident diabetes in subjects with prediabetes
Source: Cardiovasc Diabetol. 2022 Feb 7;21:21. doi: 10.1186/s12933-022-01452-5 (PMC8822824; doi:10.1186/s12933-022-01452-5)
Supplement: Supplementary file 1 — Additional file 1. Additional Tables. [file 12933_2022_1452_MOESM1_ESM.docx]

**SUPPLEMENTAL MATERIAL**

**Plasma apolipoprotein concentrations and incident diabetes in subjects with prediabetes**

Mikaël Croyal*, Matthieu Wargny*, Kevin Chemello, Chloé Chevalier, Valentin Blanchard, Edith Bigot-Corbel, Gilles Lambert, Cédric Le May, Samy Hadjadj, and Bertrand Cariou

**Supplemental Table S1** Baseline apolipoprotein plasma concentrations and the risk of new-onset diabetes during follow-up in subjects without statin treatment at baseline: Univariate logistic regression analyses

| **Apolipoproteins** | **All**  **(n = 237)** | **No diabetes**  **(n_1_ = 150)** | **New-onset diabetes**  **(n_2_ = 87)** | **OR (95% CI)** | ***p*-value** | **Data available**  **(n_1_/n_2_)** |
| --- | --- | --- | --- | --- | --- | --- |
| ApoA-I (mg/dL) | 122 [105; 142] | 122 [105; 143] | 122 [104; 138] | 0.94 (0.72–1.22) | 0.6340 | 150/87 |
| ApoA-II (mg/dL) | 22.7 [18.5; 27.5] | 22.5 [18.5; 27.4] | 22.7 [18.4; 27.8] | 1.02 (0.79–1.33) | 0.8568 | 150/87 |
| ApoA-IV (mg/dL) | 8.37 [6.32; 10.48] | 8.3 [6.59; 10.18] | 8.61 [6.02; 11.07] | 1.03 (0.79–1.35) | 0.8142 | 150/87 |
| ApoB100 (mg/dL) | 57.1 [44.5; 71.9] | 56.8 [44.6; 72.5] | 59.1 [44.7; 70.3] | 1.08 (0.83–1.41) | 0.5562 | 150/87 |
| ApoC-I (mg/dL) | 1.13 [0.93; 1.41] | 1.12 [0.93; 1.36] | 1.21 [0.94; 1.54] | 1.19 (0.91–1.55) | 0.1970 | 150/87 |
| **ApoC-II (mg/dL)** | **2.55 [1.91; 3.43]** | **2.42 [1.88; 3.35]** | **2.80 [2.05; 3.58]** | **1.34 (1.02–1.75)** | **0.0344** | **150/87** |
| **ApoC-III (mg/dL)** | **6.12 [4.88; 7.74]** | **5.94 [4.85; 7.29]** | **6.73 [5.04; 8.89]** | **1.36 (1.03–1.78)** | **0.0297** | **150/87** |
| ApoC-IV (mg/dL) | 0 [0; 0.13] | 0 [0; 0.13] | 0 [0; 0.11] | 1.00 (0.77–1.30) | 0.9854 | 150/87 |
| ApoD (mg/dL) | 2.85 [2.36; 3.49] | 2.91 [2.40; 3.60] | 2.79 [2.33; 3.32] | 0.83 (0.64–1.09) | 0.1801 | 150/87 |
| ApoE (mg/dL) | 7.07 [5.77; 8.70] | 6.58 [5.44; 8.46] | 7.74 [6.38; 9.14] | 1.28 (0.98–1.67) | 0.0705 | 150/87 |
| **ApoF (mg/dL)** | **0.77 [0.44; 1.14]** | **0.73 [0.43; 1.08]** | **0.90 [0.48; 1.35]** | **1.34 (1.02–1.76)** | **0.0336** | **150/87** |
| **ApoH (mg/dL)** | **4.57 [3.88; 5.43]** | **4.41 [3.86; 5.26]** | **4.77 [4.12; 5.86]** | **1.35 (1.02–1.78)** | **0.0364** | **150/87** |
| ApoJ (mg/dL) | 7.93 [6.63; 9.39] | 7.78 [6.58; 9.32] | 8.43 [6.68; 9.70] | 1.23 (0.94–1.61) | 0.1279 | 150/87 |
| **ApoL1 (mg/dL)** | **1.13 [0.94; 1.33]** | **1.10 [0.92; 1.28]** | **1.20 [0.95; 1.52]** | **1.41 (1.07–1.84)** | **0.0131** | **150/87** |
| ApoM (mg/dL) | 1.84 [1.49; 2.28] | 1.86 [1.52; 2.38] | 1.84 [1.48; 2.21] | 0.92 (0.70–1.22) | 0.5766 | 150/87 |
| Apo(a) (nmol/L) | 22.8 [0.0; 56.7] | 22.8 [0.0; 56.8] | 22.8 [0.0; 54.8] | 1.04 (0.80–1.36) | 0.7579 | 150/87 |

Concentrations are expressed as median [25^th^ percentile; 75^th^ percentile]. OR are calculated per 1 SD using univariate logistic regression models. The associated *p*-value is calculated using Wald test. OR, odds ratio; CI, confidence interval.

**Supplemental Table S2** Spearman correlations between plasma apolipoprotein concentrations and biochemical parameters of the subjects not on statin treatment

| **Apolipoproteins** | **BMI** | **WHR** | **FPG** | **HbA_1c_** | **Insulin** | **HOMA-IR** | **HOMA-β** | **Adiponectin** | **TC** | **HDL-C** | **LDL-C** | **TG** |
| --- | --- | --- | --- | --- | --- | --- | --- | --- | --- | --- | --- | --- |
| ApoA-I | -0.24*** | -0.17** | -0.03 | -0.08 | -0.23*** | -0.22** | -0.23*** | 0.26*** | 0.07 | 0.60*** | -0.09 | -0.20** |
| ApoA-II | -0.18** | 0.02 | -0.10 | 0.00 | -0.01 | 0.00 | -0.02 | -0.01 | 0.08 | 0.28*** | -0.05 | 0.03 |
| ApoA-IV | -0.17** | -0.05 | -0.13 | 0.04 | -0.14* | -0.13 | -0.16* | -0.03 | -0.03 | 0.16* | -0.08 | -0.11 |
| ApoB100 | -0.03 | 0.12 | -0.15* | 0.02 | 0.07 | 0.09 | 0.03 | -0.04 | 0.38*** | -0.16* | 0.38*** | 0.29*** |
| ApoC-I | 0.06 | 0.17* | -0.11 | 0.09 | 0.19** | 0.21** | 0.11 | -0.15* | 0.28*** | -0.07 | 0.18** | 0.36*** |
| ApoC-II | 0.01 | 0.20** | -0.09 | 0.07 | 0.09 | 0.11 | 0.02 | -0.17* | 0.39*** | -0.10 | 0.25*** | 0.49*** |
| ApoC-III | 0.10 | 0.17** | -0.08 | 0.08 | 0.21** | 0.23*** | 0.13 | -0.26*** | 0.37*** | -0.23*** | 0.28*** | 0.53*** |
| ApoC-IV | -0.03 | 0.04 | -0.03 | -0.04 | -0.06 | -0.07 | -0.07 | 0.03 | 0.05 | 0.09 | 0.04 | 0.03 |
| ApoD | -0.42*** | 0.02 | -0.08 | -0.12 | -0.34*** | -0.32*** | -0.37*** | 0.19** | 0.19** | 0.36*** | 0.12 | -0.12 |
| ApoE | 0.06 | 0.06 | -0.10 | 0.06 | 0.10 | 0.13 | 0.05 | -0.06 | 0.26*** | -0.02 | 0.14* | 0.33*** |
| ApoF | -0.10 | 0.01 | 0.02 | 0.07 | 0.08 | 0.10 | 0.04 | -0.11 | 0.02 | 0.03 | 0.00 | 0.06 |
| ApoH | 0.03 | 0.09 | -0.12 | 0.10 | 0.20** | 0.21** | 0.15* | -0.21** | 0.02 | 0.01 | -0.03 | 0.15* |
| ApoJ | 0.03 | 0.02 | -0.04 | -0.06 | 0.13 | 0.13 | 0.13* | -0.04 | -0.03 | 0.00 | -0.08 | 0.06 |
| ApoL1 | 0.04 | -0.05 | -0.05 | -0.01 | 0.16* | 0.16* | 0.15* | -0.14* | -0.01 | -0.05 | -0.10 | 0.22*** |
| ApoM | -0.19** | 0.03 | -0.11 | -0.04 | -0.09 | -0.09 | -0.11 | 0.07 | 0.17** | 0.32*** | 0.05 | 0.01 |
| Apo(a) | -0.08 | -0.03 | 0.00 | -0.02 | -0.05 | -0.05 | -0.02 | 0.07 | 0.08 | 0.02 | 0.07 | -0.08 |

N = 237. Values are expressed as Spearman’s correlation coefficients (95% CI). *, *p* < 0.05; **, *p* < 0.01; ***, *p* < 0.001. BMI, body mass index; WHR, waist/hip circumference ratio; FPG, fasting plasma glucose; HOMA-IR/-β, homeostasis model assessment of insulin resistance/β-cell function; TC, total cholesterol; HDL-C, high-density lipoprotein cholesterol; LDL-C, low-density lipoprotein cholesterol; TG, triglycerides.

**Supplemental Table S3** Association between plasma apolipoprotein concentrations at baseline and the incidence of diabetes during follow-up (Cox models based on the proportional hazards assumption)

|  | **Model 1** | | **Model 2** | | **Model 3** | | **Model 4** | |
| --- | --- | --- | --- | --- | --- | --- | --- | --- |
|  | **HR (95% CI)** | ***p*-value** | **HR (95% CI)** | ***p*-value** | **HR (95% CI)** | ***p*-value** | **HR (95% CI)** | ***p*-value** |
| **Adjustment factors (baseline values)** |  |  |  |  |  |  |  |  |
| Age | 0.99 [0.82; 1.20] | 0.95 | - | - | - | - | - | - |
| Sex (female/male) | 1.21 [0.82; 1.79] | 0.34 | - | - | - | - | - | - |
| **Body mass index** | **1.39 [1.19; 1.62]** | **<0.0001** | - | - | - | - | - | - |
| **Fasting plasma glucose** | **1.43 [1.19; 1.71]** | **0.0001** | - | - | - | - | - | - |
| **HbA_1c_** | **1.78 [1.45; 2.18]** | **<0.0001** | - | - | - | - | - | - |
| **Plasma lipids** |  |  |  |  |  |  |  |  |
| Total cholesterol | 0.92 [0.76; 1.10] | 0.35 | 1.02 [0.85; 1.24] | 0.82 | 1.04 [0.85; 1.28] | 0.70 | - | - |
| **Triglycerides** | **1.21 [1.03; 1.43]** | **0.024** | 1.20 [0.99; 1.45] | 0.06 | 1.21 [1.00; 1.47] | 0.052 | - | - |
| LDL-C | 0.93 [0.77; 1.12] | 0.43 | 1.02 [0.84; 1.24] | 0.86 | 1.04 [0.83; 1.29] | 0.75 | - | - |
| **HDL-C** | **0.80 [0.66; 0.97]** | **0.025** | 0.89 [0.71; 1.11] | 0.29 | 0.88 [0.70; 1.10] | 0.26 | - | - |
| Non-HDL-C | 1.00 [0.83; 1.20] | 0.97 | 1.06 [0.87; 1.28] | 0.57 | 1.08 [0.88; 1.33] | 0.44 | - | - |

**Supplemental Table S3** Continued, end.

|  | | **Model 1** | | | | **Model 2** | | | | **Model 3** | | | **Model 4** | |
| --- | --- | --- | --- | --- | --- | --- | --- | --- | --- | --- | --- | --- | --- | --- |
|  | | **HR (95% CI)** | | ***p*-value** | | **HR (95% CI)** | | ***p*-value** | | **HR (95% CI)** | | ***p*-value** | **HR (95% CI)** | ***p*-value** |
| **Plasma apolipoproteins** | |  | |  | |  | |  | |  |  |  |  |  |
| ApoA-I | | 0.94 [0.78; 1.14] | | 0.54 | | 1.07 [0.88; 1.30] | | 0.51 | | 1.07 [0.87; 1.31] | 0.52 | 1.09 [0.89; 1.34] | 0.39 |  |
| Apo-II | | 1.06 [0.88; 1.29] | | 0.54 | | 1.17 [0.96; 1.43] | | 0.13 | | 1.16 [0.94; 1.42] | 0.16 | 1.13 [0.92; 1.39] | 0.24 |  |
| ApoA-IV | | 0.95 [0.79; 1.13] | | 0.55 | | 0.96 [0.80; 1.15] | | 0.62 | | 0.96 [0.80; 1.14] | 0.62 | 0.95 [0.79; 1.14] | 0.59 |  |
| ApoB100 | | 0.98 [0.80; 1.19] | | 0.82 | | 1.03 [0.84; 1.25] | | 0.79 | | 1.05 [0.86; 1.28] | 0.65 | 0.97 [0.78; 1.20] | 0.78 |  |
| ApoC-I | | 1.15 [0.97; 1.37] | | 0.10 | | **1.20 [1.01; 1.43]** | | **0.041** | | **1.21 [1.01; 1.44]** | **0.036** | 1.14 [0.92; 1.41] | 0.22 |  |
| **ApoC-II** | | **1.26 [1.06; 1.51]** | | **0.010** | | **1.28 [1.07; 1.53]** | | **0.008** | | **1.28 [1.07; 1.54]** | **0.007** | 1.24 [0.99; 1.57] | 0.06 |  |
| **ApoC-III** | | **1.23 [1.04; 1.46]** | | **0.019** | | **1.22 [1.02; 1.46]** | | **0.031** | | **1.23 [1.03; 1.48]** | **0.024** | 1.17 [0.93; 1.47] | 0.18 |  |
| ApoC-IV | | 0.97 [0.67; 1.41] | | 0.88 | | 1.05 [0.72; 1.53] | | 0.79 | | 1.06 [0.73; 1.55] | 0.75 | 1.04 [0.71; 1.52] | 0.85 |  |
| ApoD | | 0.94 [0.79; 1.13] | | 0.52 | | 1.12 [0.92; 1.36] | | 0.28 | | 1.11 [0.91; 1.36] | 0.30 | 1.15 [0.94; 1.41] | 0.17 |  |
| **ApoE** | | **1.24 [1.06; 1.47]** | | **0.009** | | **1.29 [1.10; 1.51]** | | **0.002** | | **1.29 [1.10; 1.51]** | **0.002** | **1.28 [1.06; 1.54]** | **0.010** |  |
| **ApoF** | | **1.24 [1.02; 1.49]** | | **0.029** | | **1.22 [1.01; 1.48]** | | **0.038** | | **1.22 [1.01; 1.48]** | **0.035** | **1.22 [1.01; 1.48]** | **0.037** |  |
| **ApoH** | | **1.22 [1.02; 1.45]** | | **0.029** | | 1.21 [1.00; 1.46] | | 0.053 | | **1.22 [1.01; 1.48]** | **0.042** | 1.20 [0.98; 1.45] | 0.07 |  |
| **ApoJ** | | 1.19 [0.99; 1.42] | | 0.065 | | **1.24 [1.04; 1.49]** | | **0.018** | | **1.25 [1.04; 1.50]** | **0.016** | **1.24 [1.03; 1.49]** | **0.024** |  |
| **ApoL1** | | **1.31 [1.10; 1.56]** | | **0.003** | | **1.30 [1.09; 1.55]** | | **0.004** | | **1.29 [1.08; 1.54]** | **0.005** | **1.26 [1.05; 1.52]** | **0.014** |  |
| ApoM | | 0.94 [0.77; 1.16] | | 0.59 | | 1.02 [0.84; 1.24] | | 0.84 | | 1.02 [0.84; 1.24] | 0.85 | 1.00 [0.82; 1.22] | 1 |  |
| Apo(a) | | 0.97 [0.80; 1.17] | | 0.74 | | 0.93 [0.77; 1.12] | | 0.44 | | 0.92 [0.76; 1.12] | 0.41 | 0.91 [0.75; 1.10] | 0.34 |  |

For quantitative parameters (i.e., all parameters except ApoC-IV), hazard ratios (HRs) are calculated per 1 SD after natural log transformation. Boldface indicates significance at *p* ≤ 0.05. Model 1: not adjusted (univariate); model 2: adjusted for baseline values of age, sex, body mass index, fasting plasma glucose, and HbA_1c_; model 3: model 2 + adjustment for the use of statins or fibrates; model 4: model 3 + triglycerides.

**Supplemental Table S4** Distribution of apoE phenotypes according to the diabetes status at the end of the follow-up

| **ApoE phenotypes** | **All subjects**  **(n = 307)** | **No diabetes**  **(n = 192)** | **New-onset diabetes**  **(n = 115)** | ***p*-value** |
| --- | --- | --- | --- | --- |
| ApoE2/E2 | 1 (0.3) | 0 (0.0) | 1 (0.9) | 0.67 |
| ApoE2/E3 | 31 (10.1) | 18 (9.4) | 13 (11.3) |  |
| ApoE2/E4 | 9 (2.9) | 5 (2.6) | 4 (3.5) |  |
| ApoE3/E3 | 192 (62.5) | 120 (62.5) | 72 (62.6) |  |
| ApoE3/E4 | 74 (24.1) | 49 (25.5) | 25 (21.7) |  |
| ApoE4/E4 | 0 (0.0) | 0 (0.0) | 0 (0.0) |  |

Data are presented as the frequency (%) for each category. The *p*-value was calculated using Fisher’s exact test.
